# Fewer subsequent relapses and lower levels of IL-17 in Takayasu arteritis developed after the age of 40 years

**DOI:** 10.1186/s13075-016-1193-9

**Published:** 2016-12-13

**Authors:** Shoichi Fukui, Naoki Iwamoto, Toshimasa Shimizu, Masataka Umeda, Ayako Nishino, Tomohiro Koga, Shin-ya Kawashiri, Kunihiro Ichinose, Yasuko Hirai, Mami Tamai, Hideki Nakamura, Toshiyuki Aramaki, Nozomi Iwanaga, Yasumori Izumi, Tomoki Origuchi, Kiyoshi Migita, Yukitaka Ueki, Shuntaro Sato, Atsushi Kawakami

**Affiliations:** 1 Unit of Advanced Preventive Medical Sciences, Departments of Immunology and Rheumatology, Nagasaki University Graduate School of Biomedical Sciences, 1-7-1 Sakamoto, Nagasaki, 852-8501 Japan; 2 Unit of Advanced Preventive Medical Sciences, Departments of Community Medicine, Nagasaki University Graduate School of Biomedical Sciences, Nagasaki, Japan; 3 Rheumatic and Collagen Disease Center, Sasebo Chuo Hospital, Sasebo, Nagasaki Japan; 4 Department of General Internal Medicine and Rheumatology, Clinical Research Center, NHO Nagasaki Medical Center, Omura, Nagasaki Japan; 5 Unit of Rehabilitation Sciences, Department of Locomotive Rehabilitation Science, Nagasaki University Graduate School of Biomedical Sciences, Nagasaki, Japan; 6 Nagasaki University Hospital Clinical Research Center, Nagasaki, Japan

**Keywords:** Age, Chemokines, Cytokines, IL-17, Relapse, Takayasu arteritis

## Abstract

**Background:**

The clinical characteristics of Takayasu arteritis (TAK) developing in individuals older than 40 years (TAK >40) are little-known.

**Method:**

We retrospectively analyzed 43 patients with TAK treated at three hospitals in Japan from April 2000 to March 2016. From medical records we collected baseline variables at diagnosis including clinical symptoms, laboratory data, and subsequent relapses. We compared these indices in the patients with TAK onset at >40 years of age (TAK >40) to those with TAK onset ≤40 years (TAK ≤40). Multiplex cytokine/chemokine bead assays were performed using preserved serum supernatants from 24 patients with TAK and 40 healthy donors.

**Results:**

Of the 43 patients, 20 had TAK >40; this group had significantly fewer instances of orthostatic hypotension (2 (10%) vs. 10 (43%), *p* = 0.019), carotid bruit (7 (35%) vs. 16 (70%), *p* = 0.034), and chest pain (0 (0%) vs. 6 (26%), *p* = 0.023) compared to patients with TAK ≤40 (n = 23). The initial prednisolone dose was significantly lower in TAK >40 (median 30 mg vs. 40 mg per day, *p* = 0.024). Assessed by the log-rank test, the relapse-free survival rate after remission was significantly higher in the patients with TAK >40 (*p* = 0.029). The interleukin 17 levels were significantly lower in patients with TAK >40 compared to patients with TAK ≤40 and healthy donors.

**Conclusion:**

Compared to TAK ≤40, TAK >40 could be treated by lower initial doses of prednisolone to achieve remission, and with fewer relapses. These differences might be due to the difference of T helper 17 (Th17) activity suggested by the cytokine profiles.

## Background

Takayasu arteritis (TAK) is a type of large-vessel vasculitis that predominantly affects young women [1]. In general, TAK is diagnosed based on The American College of Rheumatology (ACR) 1990 criteria for the classification of Takayasu arteritis [2], which consist of the following six components: (1) age at disease onset ≤40 years (TAK ≤40); (2) claudication in the extremities; (3) decreased brachial artery pulse; (4) difference between arms in systolic blood pressure of 10 mmHg; (5) bruit over the subclavian arteries or the aorta; and (6) abnormality on arteriogram. Patients with at least three of these components are classified as having TAK. When these ACR criteria were used in a previous study, of 63 patients of TAK, there were only 4 with onset of TAK >40 years of age (TAK >40) [2]. In contrast, in a French study, 32% of patients with TAK had TAK > 40 [3], which suggests that it is not rare to be diagnosed with TAK >40 years of age.

The patient’s age at diagnosis is important in autoimmune diseases. For example, the clinical characteristics of patients with elderly-onset rheumatoid arthritis (RA) are different from those of younger patients with RA [4]. Similarly, age at diagnosis is important in distinguishing TAK from giant cell arteritis (GCA), because both TAK and GCA are categorized as forms of large-vessel vasculitis; in addition, the ACR 1990 criteria for the classification of GCA [5] include the component ‘age at disease onset ≥50 years.’ The boundaries between TAK and GCA have become increasingly blurred in the clinical aspects of these conditions [6]. Therefore, age at diagnosis or at disease onset is very important.

Ohigashi et al. reported the clinical characteristics of patients with TAK >40; these patients had significantly more coronary artery disease and hypertension, and significantly fewer instances of aortic regurgitation [7]. However, except for this study, little is known about the relationship between the age of disease onset and clinical characteristics of TAK.

Here we attempted to identify the clinical differences, including relapse and prognosis, in patients with TAK >40 and patients with TAK ≤40. In addition, we evaluated differences in the profile of serum cytokines and chemokines at diagnosis between TAK >40 and TAK ≤40.

## Methods

### Patients and study criteria

**Fig. 1:**
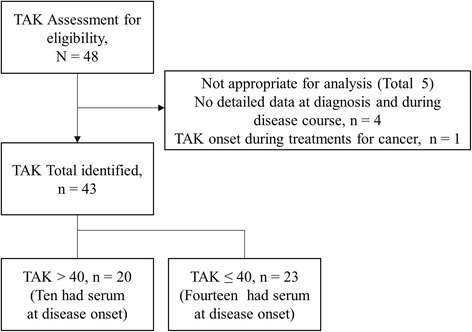
Flow diagram: 43 patients with Takayasu arteritis (*TAK*) were included in the study

We retrospectively analyzed all patients with TAK who were registered and followed by the Nagasaki University Hospital, Sasebo Chuo Hospital, and NHO Nagasaki Medical Center between April 2000 and March 2016. We included patients with diagnosis based on the ACR 1990 criteria for the classification of TAK [2]. Four patients were excluded because detailed data were missing at diagnosis and/or during the disease course. One patient was excluded because she developed TAK during chemotherapy for ovarian cancer. A final total of 43 patients were included this study (Fig. 1). They were all newly diagnosed with TAK and were followed up for at least 6 months. No enrolled patients fulfilled the ACR 1990 criteria for the classification of GCA [5].

To compare the characteristics of TAK among patients of different age at disease onset, we divided the 43 patients by age at disease onset: >40 years old (the TAK >40 group) and ≤40 years old at disease onset (the TAK ≤40 group). None of the TAK >40 patients had history of symptoms of TAK 40 years of age. TAK >40 fulfilled three or more items in the ACR criteria for TAK without the definition of onset of age. TAK ≤40 also fulfilled three or more items, including age.

Relapse was determined by Japan College of Rheumatology-certified rheumatologists by confirmation of the recurrence of active disease as described [8]. The criteria for relapse were the detection of a new vascular lesion or lesions (aneurysms, stenoses or occlusions, or new irregularities of the arterial wall) in arteries (aorta, innominate, subclavian, axillary, common and internal carotid, vertebral, superior and inferior mesenteric, renal, common iliac, or celiac axis) on angiography (including computed tomography) in patients who had undergone angiography before being evaluated for relapse, or at least two of the following: (1) new onset of carotidynia or pain over the large vessels; (2) transient ischemic episodes not attributable to other factors; (3) new bruit or new asymmetry in pulses or blood pressure determination; (4) ischemic symptoms (including new-onset claudication); and (5) fever in the absence of infection. Angiography was performed when the patient’s attending doctor considered it necessary on the basis of the patient’s clinical characteristics. We recruited healthy donors (HD) from the staff of Nagasaki University.

### Clinical and laboratory analysis

We collected baseline information at diagnosis, including clinical characteristics and laboratory data and subsequent relapses, by accessing the patients’ medical records. The baseline clinical characteristics at diagnosis included age, sex, symptoms, body mass index (BMI), history of smoking, angiographic classification types, and complications including diabetes mellitus (DM) and chronic kidney disease (CKD) (these complications influence vascular conditions), inflammatory bowel diseases (IBD), and pyoderma gangrenosum (PG) (TAK is frequently complicated with IBD or PG).

The initial laboratory data included human leukocyte antigen (HLA) typing, white blood cell count, hemoglobin, platelet count, C reactive protein (CRP), erythrocyte sedimentation rate (ESR), albumin (Alb), and immunoglobulin G (IgG). Baseline information at diagnosis was collected within 2 weeks before treatment started, except for the HLA typing.

We analyzed the corticosteroid doses received in both the initial treatment and on relapse in the 43 patients with TAK who had been followed up for ≥6 months. Information on the use of pulsed methylprednisolone and immunosuppressants including methotrexate, cyclosporine, and tacrolimus was also collected, as was information on the use of biologics including infliximab, adalimumab, and tocilizumab. The corticosteroid doses were calculated as daily prednisolone equivalents. The rate of reduction in the prednisolone dose (mg/month) was calculated using the reduction dose during the 6 months after starting corticosteroid treatment. In particular, we subtracted the prednisolone dose at 6 months after remission from the initial prednisolone dose and we divided the difference by 6 months.

### Multiplex cytokine/chemokine bead assays

Multiplex cytokine/chemokine bead assays were performed using diluted serum supernatants and Milliplex MAP Human Cytokine/Chemokine Panel 1 Pre-mixed 41Plex (Merck Millipore, Darmstadt, Germany) analyzed with a Bio-Plex® MAGPIX™ Multiplex Reader (Bio-Rad, Hercules, CA, USA) according to the manufacturers’ instructions. The cytokines/chemokines measured by the Milliplex MAP Human Cytokine/Chemokine Panel 1 Pre-mixed 41Plex included vascular endothelial growth factor (VEGF), TNF-β, TNF-α, transforming growth factor (TGF)-α, regulated and normal T cell expressed and secreted (RANTES), platelet-derived growth factor (PDGF)-AB/BB, PDGF-AA, macrophage inflammatory protein (MIP)-1β, MIP-1α, myeloid dendritic cells (MDC) (CCL22), monocyte chemotactic protein (MCP)-3, MCP-1, IP-10, IL-17, IL-15, IL-13, IL-12 (p70), IL-12 (p40), IL-10, IL-9, IL-8, IL-7, IL-6, IL-5, IL-4, IL-3, IL-2, IL-1ra, IL-1β, IL-1α, interferon (IFN)-γ, IFN-α2, growth-related cytokine (GRO), granulocyte macrophage-colony stimulating factor (GM-CSF), granulocyte-colony stimulating factor (G-CSF), fractalkine, Flt-3 ligand, fibroblast growth factor (FGF)-2, eotaxin, epidermal growth factor (EGF), and sCD40L.

Serum obtained from patients with TAK at diagnosis and from HD was centrifuged within 30 minutes at 1500 rpm at 4 °C for 5 minutes, and the liquid phase serum was stored at −80 °C until use. Serum from HD was divided into two analysis groups of HD older than 40 years (HD >40) and HD who were ≤40 years old (HD ≤40). We compared serum from HD with serum from patients with TAK >40 and TAK ≤40. Serum was obtained from HD in 2013–2015. The earliest obtained serum from a patient at diagnosis was 2009. Multiplex cytokine/chemokine bead assays using serum from patients and HD were performed in 2015–2016.

### Statistical analysis

We compared the baseline characteristics, including demographic data, hematologic data, serum markers, treatments, and follow-up duration between the TAK >40 and TAK ≤40 groups. Variables are described using frequencies for categorical variables and with the median and interquartile range (IQR) for quantitative variables. We assessed the association between variables using Fisher’s exact test for categorical variables and Wilcoxon’s rank sum test for quantitative variables. The relapse-free survival rate after remission was estimated by the Kaplan-Meier method. The association between the relapse-free survival rate and TAK >40 or TAK ≤40 was evaluated by the log-rank test. All tests were two-sided and a *p* value <0.05 was considered significant. The Steel-Dwass test was performed for comparisons of multiplex cytokine/chemokine bead assay results in the four groups, i.e., TAK >40, TAK ≤40, HD >40, and HD ≤40. All statistical analyses were performed using JMP Statistical Software, ver. 11 (SAS Institute, Cary, NC, USA) and GraphPad Prism ver. 7.0 (GraphPad Software, San Diego, CA, USA).

## Results

### Baseline characteristics

The study population was 43 patients who were identified over the 16-year period. Twenty of the patients with TAK were classified as TAK >40. Table 1 shows the demographic, clinical and laboratory characteristics of the TAK >40 and TAK ≤40 (*n* = 23) groups. Median age in the TAK >40 and TAK ≤40 groups was 56 and 23 years, respectively. The positive rate of HLA-B52, the rate of complications, and angiographic classification were not significantly different between the two groups.

**Table 1:** Demographic, clinical, and laboratory characteristics of patients in the TAK >40 and TAK ≤40 groups

Variable	TAK >40, *n* = 20	TAK ≤40, *n* = 23	*P* value
Onset age (IQR)	56 (48–62)	23 (20–28)	
Female, *n* (%)	19 (95)	21 (91)	1.00
HLA-B52, *n* (%)	7 (47) , *n* = 15	11 (65), *n* = 17	0.48
BMI, kg/m^2^ (IQR)	21.9 (20.2–26.2)	21.2 (18.9–22.7)	0.55
Family history	1 (5)	0 (0)	0.47
Smoking	5 (25)	4 (17)	0.71
Complication, *n* (%)			
DM	2 (10)	2 (9)	1.00
CKD	0 (0)	2 (9)	0.49
IBD	1 (5)	2 (9)	1.00
PG	1 (5)	1 (4)	1.00
Angiographic classification, *n* (%)			
Type I	5 (25)	5 (22)	1.00
Type IIa	2 (10)	3 (13)	1.00
Type IIb	3 (15)	4 (17)	1.00
Type IV	1 (5)	0 (0)	0.47
Type V	9 (45)	11 (48)	1.00
Fever	11 (55)	14 (61)	0.76
Malaise	10 (50)	12 (52)	1.00
Headache	5 (25)	12 (52)	0.118
Dizziness	0 (0)	5 (22)	0.051
Orthostatic hypotension	2 (10)	10 (43)	0.019*
Ophthalmic symptoms	1 (5)	4 (17)	0.35
Carotid bruit	7 (35)	16 (70)	0.034*
Cervical pain	4 (20)	6 (26)	0.73
Discrepancy of blood pressure	10 (50)	11 (48)	1.00
Upper limb pain	4 (20)	9 (39)	0.20
Pulselessness	4 (20)	6 (26)	0.73
Chest pain	0 (0)	6 (26)	0.023*
Abdominal bruit	6 (30)	6 (26)	1.00
Arthralgia	5 (25)	6 (26)	1.00
Aortic valve regurgitation	5 (25)	7 (30)	0.74
WBC, ×103/μL (normal range 3.5–9.8) (IQR)	9.4 (7.1–11.7), *n* = 20	8.2 (6.6–10.3), *n* = 23	0.56
Hb, g/dL (normal range11.3–17.6) (IQR)	12.4 (10.1–13.2), *n* = 20	10.8 (9.4–12.0), *n* = 23	0.054
Plt × 104/μL, (normal range 13.0–36.9) (IQR)	29.3 (23.4–38.2), *n* = 20	35.1 (25.1–47.0), *n* = 23	0.28
CRP, mg/dL (normal range <0.14) (IQR)	4.2 (0.5–9.0), *n* = 20	7.0 (0.6–9.6), *n* = 23	0.62
ESR, mm/h, (normal range <15) (IQR)	75 (32–91), *n* = 18	77 (27–106), *n* = 17	0.74
Alb, g/dL, (normal range 3.8–5.2) (IQR)	3.7 (3.1–4.1), *n* = 19	3.6 (3.2–4.3), *n* = 21	0.95
IgG, mg/dL, (normal range 870–1700) (IQR)	1410 (1100–1670), *n* = 17	1660 (1190–2190), *n* = 22	0.33

*TAK >40* Takayasu arteritis onset after age 40 years; *TAK ≤40* TAK onset age 40 years or younger, *BMI* body mass index, *DM* diabetes mellitus, *CKD* chronic kidney disease, *IBD* inflammatory bowel diseases, *PG* pyoderma gangrenosum, *WBC* white blood cell count, *Hb* hemoglobin, *Plt* platelet, *CRP* C reactive protein, *ESR* erythrocyte sedimentation rate, *Alb* albumin, *IgG* immunoglobulin. *p < 0.05

In contrast, the TAK >40 group had significantly lower rates of orthostatic hypotension (2 (10%) vs. 10 (43%), *p* = 0.019), carotid bruit (7 (35%) vs. 16 (70%), *p* = 0.034) and chest pain (0 (0%) vs. 6 (26%), *p* = 0.023) at diagnosis compared to the TAK ≤40 group. There were no significant between-group differences in CRP or ESR. Furthermore, because we could not eliminate the potential bias in the diagnosis of TAK by using the criterion of age at disease onset, we extracted TAK ≤40 who fulfilled at least three of the five criteria excluding the criterion of age at disease onset from the whole TAK ≤40 group and then compared these patients with the TAK >40 group (Table 2). In the TAK ≤40 group, 14 out of 23 patients fulfilled at least three of the five criteria excluding the criterion of age at disease onset. Patients with TAK ≤40 who fulfilled at least three criteria excluding the age criterion had significantly more headaches, dizziness, orthostatic hypotension, upper limb pain, chest pain and lower hemoglobin levels compared to the TAK >40 group; however, there were largely no differences as compared with the data in Table 1.

**Table 2:** Demographic, clinical, and laboratory characteristics of patients in the TAK >40 and TAK ≤40 groups who fulfilled at least three criteria, excluding the age criterion

Variable	TAK >40, *n* = 20	TAK ≤40 who fulfilled at least three criteria, excluding the age criterion, *n* = 14	*P* value
Onset age, years (IQR)	56 (48–62)	23 (20–29)	
Female, *n* (%)	19 (95)	13 (93)	1.00
HLA-B52, *n* (%)	7 (47) , *n* = 15	10 (83), *n* = 12	0.107
BMI, kg/m^2^ (IQR)	21.9 (20.2–26.2)	21.2 (18.6–22.2)	0.48
Family history	1 (5)	0 (0)	1.00
Smoking	5 (25)	3 (21)	1.00
Complication, *n* (%)			
DM	2 (10)	1 (7)	1.00
CKD	0 (0)	1 (7)	0.41
IBD	1 (5)	1 (7)	1.00
PG	1 (5)	1 (7)	1.00
Angiographic classification, *n* (%)			
Type I	5 (25)	5 (36)	0.70
Type IIa	2 (10)	3 (21)	0.63
Type IIb	3 (15)	2 (14)	1.00
Type IV	1 (5)	0 (0)	1.00
Type V	9 (45)	4 (29)	0.48
Fever	11 (55)	10 (71)	0.48
Malaise	10 (50)	8 (57)	0.74
Headache	5 (25)	9 (64)	0.035*
Dizziness	0 (0)	4 (29)	0.022*
Orthostatic hypotension	2 (10)	8 (43)	0.006*
Ophthalmic symptoms	1 (5)	4 (29)	0.135
Carotid bruit	7 (35)	10 (71)	0.080
Cervical pain	4 (20)	4 (29)	0.69
Discrepancy in blood pressure	10 (50)	10 (71)	0.30
Upper limb pain	4 (20)	8 (57)	0.036*
Pulselessness	4 (20)	6 (43)	0.25
Chest pain	0 (0)	4 (29)	0.022*
Abdominal bruit	6 (30)	4 (29)	1.00
Arthralgia	5 (25)	3 (21)	1.00
Aortic valve regurgitation	5 (25)	7 (50)	0.16
WBC, ×103/μL (normal range 3.5–9.8) (IQR)	9.4 (7.1–11.7), *n* = 20	9.9 (7.1–14.3), *n* = 14	0.66
Hb, g/dL (normal range11.3–17.6) (IQR)	12.4 (10.1–13.2), *n* = 20	10.8 (8.8–11.7), *n* = 14	0.033*
Plt × 104/μL, (normal range 13.0–36.9) (IQR)	29.3 (23.4–38.2), *n* = 20	36.9 (24.2–51.7), *n* = 14	0.26
CRP, mg/dL (normal range <0.14) (IQR)	4.2 (0.5–9.0), *n* = 20	7.0 (0.9–10.0), *n* = 14	0.46
ESR, mm/h, (normal range <15) (IQR)	75 (32–91), *n* = 18	75 (50–88), *n* = 10	0.85
Alb, g/dL, (normal range 3.8–5.2) (IQR)	3.7 (3.1–4.1), *n* = 19	3.7 (3.1–4.4), *n* = 13	0.95
IgG, mg/dL, (normal range 870–1700) (IQR)	1410 (1100–1670), *n* = 17	1660 (1170–2250), *n* = 14	0.34

*TAK >40* Takayasu arteritis onset after age 40 years; *TAK ≤40* TAK onset age 40 years or younger, *BMI* body mass index, *DM* diabetes mellitus, *CKD* chronic kidney disease, *IBD* inflammatory bowel diseases, *PG* pyoderma gangrenosum, *WBC* white blood cell count, *Hb* hemoglobin, *Plt* platelet, *CRP* C reactive protein, *ESR* erythrocyte sedimentation rate, *Alb* albumin, *IgG* immunoglobulin. *P < 0.05

### Treatments

Table 3 shows the characteristics of the treatments administered to the TAK >40 and TAK ≤40 groups. The initial prednisolone dose was significantly lower in the TAK >40 compared to the TAK ≤40 group (median 30 mg vs. 40 mg per day, respectively, *p* = 0.024). The initial prednisolone dose per kg of body weight was also significantly lower in the TAK >40 compared to the TAK ≤40 group (0.55 mg/kg per day vs. 0.80 mg/kg per day, *p* = 0.048). There were no significant between-group differences in the other immunosuppressants. Five patients in the TAK ≤40 group were treated with infliximab compared to none of the patients in the TAK >40 group. There were no significant between-group differences in the rate of reduction in the dose of prednisolone or follow-up duration.

**Table 3:** Treatments and follow-up duration in the TAK >40 and TAK ≤40 groups

Variable	TAK >40, *n* = 20	TAK ≤40, *n* = 23	*P* value
Initial dose of prednisolone, mg/day (IQR)	30 (15–40)	40 (30–45)	0.024*
Initial dose of prednisolone, mg/day (IQR)	0.55 (0.28–0.82)	0.80 (0.59–0.94)	0.048*
Pulsed methylprednisolone as an initial therapy	2 (10)	7 (30)	0.142
Methotrexate with prednisolone before relapses, *n* (%), mg/week (IQR)	7 (35), 8 (6–8)	9 (39), 10 (7–13)	1.00
Cyclosporine with prednisolone before relapses, *n* (%), mg/day (IQR)	1 (5), 150	4 (17), 160 (105–238)	0.35
Tacrolimus with prednisolone before relapses, *n* (%), mg/day (IQR)	1 (5), 3.0	1 (4), 0.5	1.00
Infliximab, *n* (%)	0 (0)	5 (22)	0.051
Adalimumab, *n* (%)	0 (0)	1 (4)	1.00
Tocilizumab, *n* (%)	1 (5)	1 (4)	1.00
Reduction rate of prednisolone dose, mg/month (IQR)	2.7 (2.3–3.0)	2.8 (2.4–2.9)	0.87
Dose of prednisolone at relapse, mg/day (IQR)	10 (8–16)	15 (11–20)	0.11
Follow-up duration, months (IQR)	70 (25–133)	55 (28–115)	0.94

*TAK >40* Takayasu arteritis onset after age 40 years; *TAK ≤40* TAK onset at age 40 years or younger. *P < 0.05

### Outcomes

**Fig. 2:**
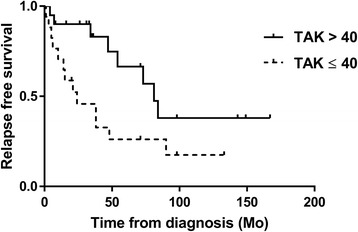
Patients with onset of Takayasu arteritis (*TAK*) at age >40 years had significantly fewer relapses as assessed by the log-rank test (*p* = 0.029) using the Kaplan-Meier method. *TAK > 40* TAK onset after age 40 years, *TAK ≤40* TAK onset age 40 years or younger

We evaluated the relapse-free survival rates in the TAK >40 and TAK ≤40 groups, and observed that the TAK >40 patients had significantly fewer relapses as assessed by the log-rank test (*p* = 0.029) using the Kaplan-Meier method (Fig. 2). There was no significant difference in surgical intervention (*p* = 0.68), and only one patient died, due to pancreatic cancer.

### Multiplex cytokine/chemokine bead assay

**Fig. 3:**
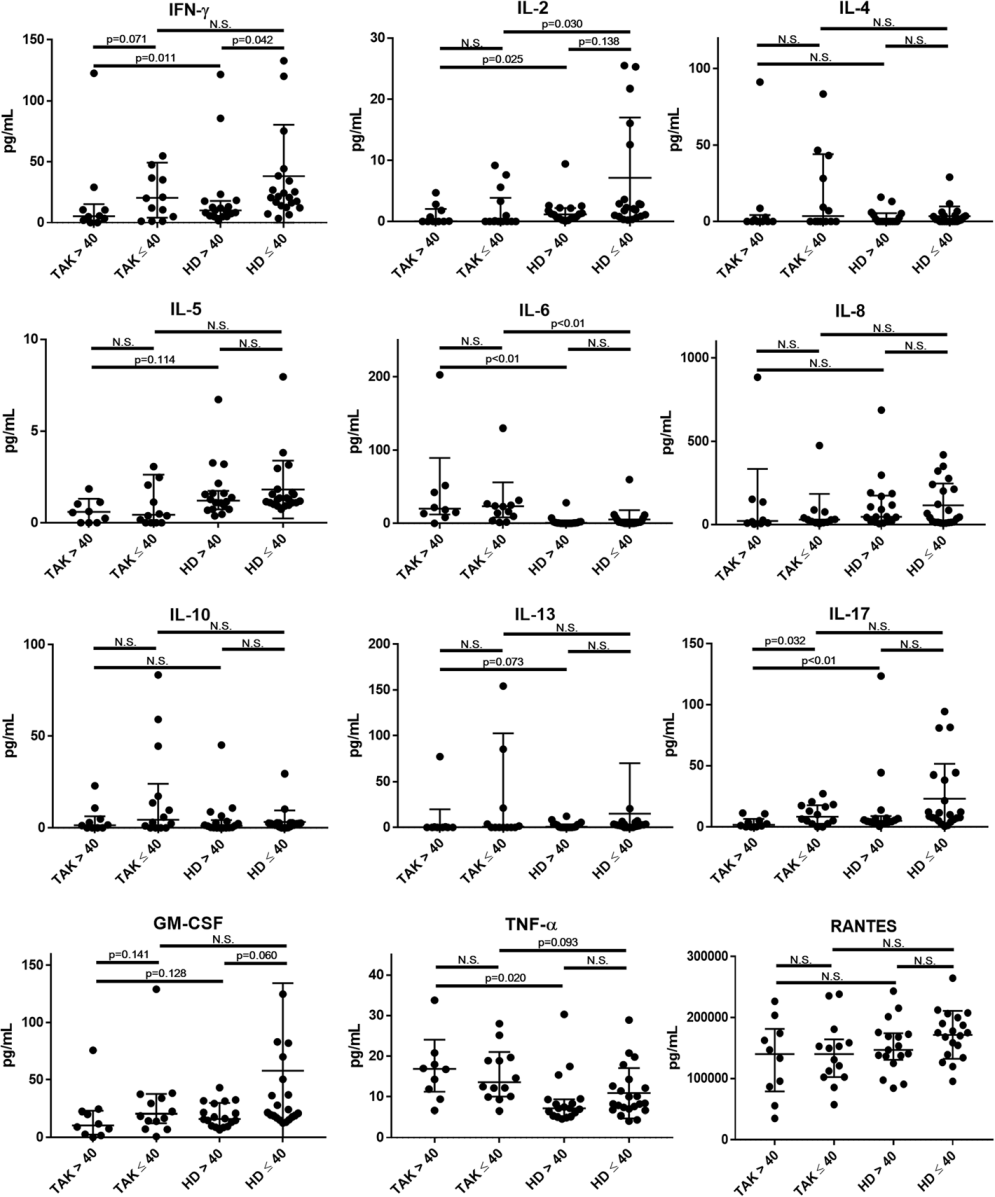
Results of a multiplex cytokines/chemokine bead assay using serum obtained at diagnosis from patients with Takayasu arteritis (*TAK*) with disease onset over 40 years of age (*TAK >40*, *n* = 10) and age at disease onset of 40 years or younger (*TAK ≤40*, *n* = 14). Serum from healthy donors (*HD*) older than 40 years (*HD >40*) and from HD 40 years of age or younger (*HD ≤40*) were compared to serum from the TAK >40 and TAK ≤40 groups. *Symbols* represent individual patients or donors. *Horizontal lines* show the median and interquartile range. *IFN-γ* interferon gamma, *IL* interleukin, *GM-CSF* granulocyte macrophage colony-stimulating factor, *TNF-α* tumor necrosis factor-α, *RANTES* regulated on activation normal T cell expressed and secreted, *N.S*. not significant

We performed a multiplex cytokine/chemokine bead assay using serum from patients with TAK >40 and patients with TAK ≤40 obtained at diagnosis (Fig. 3). We obtained serum from 10 patients with TAK >40, 14 patients with TAK ≤40, 18 HD >40, and 22 HD ≤40. Median age in the HD >40 and HD ≤ 40 groups was 46 years and 33.5 years (IQR 43–52 and 30–38 years), respectively.

The TAK >40 group had significantly lower IL-17 compared to the TAK ≤40 and the HD >40 groups (median (IQR) 1.1 (0.2–2.5) pg/mL for TAK >40; 8.4 (2.6–17.8) pg/mL for TAK ≤40; 5.1 (3.5–9.0) pg/mL for HD >40; and 9.4 (6.0–39.5) for HD ≤40). In the complete series of 24 patients with TAK, IL-6 was significantly higher in the HD >40 and the HD ≤40 groups (median (IQR) 26.9 (8.1–65.2) pg/mL; 23.4 (8.2–56.1) pg/mL; 0.6 (0–2.1) pg/mL; and 1.4 (0–5.5) pg/mL, respectively). TNF-α in the TAK >40 group was significantly higher than that in the HD >40 group (16.3 (11.2–22.1) pg/mL for TAK >40; 13.5 (10.0–21.0) pg/mL for TAK ≤40; 7.1 (5.3–9.4) pg/mL for HD >40; and 8.3 (6.9–13.0) pg/mL for HD ≤40 ). However, there were no significant differences in IL-6 or TNF-α between the TAK >40 and TAK ≤40 groups. There were no significant differences between the TAK >40 and TAK ≤40 groups in IFN-γ, IL-2, IL-4, IL-5, IL-8, IL-10, IL-13, GM-CSF, or RANTES (Fig. 3). There were no further significant differences between the TAK >40 and TAK ≤40 groups in the other cytokines/chemokines analyzed by bead assay.

## Discussion

The results of the present study revealed that there are some important clinical differences between patients with an onset at age >40 years and those with onset at age ≤40 years. The TAK >40 group had significantly fewer instances of orthostatic hypotension, carotid bruit and chest pain at diagnosis. These clinical manifestations may be caused by damage to the aortic arch and heart.

Individuals with TAK often experience relapses, even after remission. It was reported that 72% of patients with TAK experience multiple relapses within 6 months after the dose of prednisolone is tapered to <10 mg daily [9]. It is also reported that a >1.2 mg/month reduction rate in the dose of prednisolone predicts subsequent relapse [7]. However, the factors at diagnosis that could be used to predict subsequent relapse of TAK following remission have not previously been elucidated. Our present findings revealed that the TAK >40 group experienced fewer subsequent relapses compared to the TAK ≤40 group. This also suggests the disease characteristics of TAK differ by age. In addition, it is of note in the present study that the TAK >40 group was treated with lower initial doses of prednisolone to achieve remission compared to the TAK ≤40 group. We assume that each attending physician reduced the initial prednisolone doses in the TAK >40 group because of the risk of the adverse effects of prednisolone, such as infections in the elderly.

Previous investigations revealed that a skewed activation of T helper (Th)1 and Th17 pathways in patients with TAK contributes to the systemic and vascular manifestations [10, 11]. In patients with active TAK, the expression of IFN-γ, IL-12 and IL-17 is detected within aortic inflammatory infiltrates [10, 11], and the increased frequency of IFN-γ-producing and IL-17A-producing T cells and the increased production of Th1-related and Th17-related cytokines from peripheral blood mononuclear cells (PBMCs) has been observed [10]. Conversely, the change in Th2 or T regulatory cell (Treg) subsets may not be determined [11]. We thus attempted in the present study to determine whether the expression of cytokines/chemokines in serum are different in patients with TAK >40 compared to those with TAK ≤40.

We observed that IL-6 and TNF-α were higher in patients with TAK compared to the HD, which is compatible with previous studies using ELISA [11–13]. Our present data suggest a new insight, i.e., that patients with TAK >40 have relatively lower IL-17 compared to those with TAK ≤40. Furthermore, this study demonstrated no differences in IL-17 between the HD >40 and in HD ≤40 groups, which is compatible with a previous study showing that IL-17 does not differ between younger and older HD [14]. These observations may refute the possibility that the decline of IL-17 in patients with TAK >40 simply depends on advancing age. The present clear differences in IL-17 by age might explain the previous result, as patients with TAK in the previous study had a relatively wide age range at disease onset [11].

We observed no significant differences in Th1-related cytokines, including IFN-γ and IL-2 and Th2-related cytokines, including IL-4, IL-5, and IL-13, between the TAK >40 and TAK ≤40 groups. Glucocorticoids are a standard part of the treatment of TAK, and a previous study demonstrated a decrease in Th1-derived cytokines (e.g., IFN-γ) produced from PBMCs after glucocorticoid treatment, whereas the production of Th17-derived cytokines (e.g., IL-17A) was not inhibited [10]. Misra et al. also reported that IL-17 levels do not change with different immunosuppressive treatments [15]. Differences in IL-17 in the TAK >40 and TAK ≤40 groups, may suggest differences in the pathophysiological etiology of two groups. These data suggest that exploration of Th17 cell pathways is important to predict the disease outcome in TAK. In this regard, it is very interesting to note that the serum levels of IL-17 were low in our TAK >40 group, and that the clinical characteristics were less severe in those patients compared to patients in the TAK ≤40 group. A cross-sectional study showed a correlation between serum IL-6 and TAK disease activity [11], but this was the first report to describe an association between serum cytokines/chemokines and the outcome of TAK that may contribute to the effect of age.

By contrast, elevated serum IL-17 in patients with TAK compared to HD have been reported previously [15]. In addition, Saadoun et al. report that addition of serum from patients with active TAK to sorted CD4-positive T cells from HD in culture medium induces significant production of IL-17A, but only weak expression of IL-17A in patients with active TAK was observed within aortic inflammatory infiltrates [10]. There are some differences between these studies and ours in the characteristics of the patients included and the HD and methods, including the assay and sample handling. Furthermore, serum levels of cytokines and chemokines may not necessarily reflect what is occurring in the tissues. Therefore, it is necessary to exercise caution when considering the pathophysiology of TAK.

Our study has some limitations. First, we could not eliminate potential bias in the diagnosis of TAK by using the criterion of age at disease onset. Because TAK classification criteria include the criterion of age at disease onset ≤40 years, a prospective study is absolutely imperative to eliminate bias in the diagnosis of TAK. To partially address this problem, we also performed comparison of patients with TAK ≤40 who fulfilled at least three criteria excluding the age criterion and those with TAK >40. Second, more cases are needed to confirm these results, particularly in assessing the results of multiplex cytokine/chemokine bead assays. However, this study is valuable as a hypothesis-generating study. Third, we could not assess the T cell subpopulations using fluorescence-activated cell sorting because this was a retrospective study. We plan to analyze T cell subpopulations in patients with TAK >40 and TAK ≤40 in the future. Fourth, we used the ACR criteria to differentiate TAK from GCA and to exclude GCA. TAK and GCA appear more similar than different, based on clinical and pathological findings [16]. In contrast, Michel et al. insisted that GCA and TAK were easily definable and separable disorders, even when the typical age barrier of 40 years at onset of disease was excluded [17]. Although all patients in our cohort did not fulfil the ACR criteria for GCA, this method may be insufficient to exclude it. Fifth, as TAK usually develops insidiously with non-specific symptoms, onset at age >40 years may not correctly reflect the real onset of the disease. However, we assume that there are no differences in the latent duration to the time that subjective symptoms appear and physical changes are identified in both TAK >40 and TAK ≤40. Sixth, the number of patients in the TAK >40 group in this cohort seems high compared to previous studies. This difference may rely on differences in ethnicity and regions. Seventh, in multiplex cytokine/chemokine bead assays, the duration of preservation of serum differed. In this study, we did not find lower cytokine and chemokine levels in serum that had been preserved for longer periods. However, because the influence of degradation of cytokines and chemokines is unknown, we plan to measure cytokines and chemokines in preserved serum at various time points in our further research.

## Conclusions

Patients with TAK >40 had fewer relapses after remission compared to patients with TAK ≤40. The levels of IL-17 in the TAK >40 group at diagnosis were lower compared to those in the TAK ≤40 group. Our findings clarified the relationships between age, clinical characteristics, and profiles of cytokines and chemokines in patients with TAK.

## Abbreviations

ACR: American College of Rheumatology
Alb: Albumin
BMI: Body mass index
CKD: Chronic kidney disease
CRP: C reactive protein
DM: Diabetes mellitus
EGF: Epidermal growth factor
ESR: Erythrocyte sedimentation rate
FGF: Fibroblast growth factor
GCA: Giant cell arteritis
HD: Healthy donors
HLA: Human leukocyte antigen
IBD: Inflammatory bowel diseases
IFN: Interferon
IgG: Immunoglobulin G
IQR: Interquartile range
MDC: Myeloid dendritic cells
MCP: Monocyte chemotactive protein
MIP: Macrophage inflammatory protein
PBMCs: Peripheral blood mononuclear cells
PDGF: Platelet-derived growth factor
PG: Pyoderma gangrenosum
RA: Rheumatoid arthritis
RANTES: Regulated and normal t cell expressed and secreted
TAK: Takayasu arteritis
TGF: Transforming growth factor
TNF: Tumor necrosis factor
VEGF: Vascular endothelial growth factor
